# Adverse childhood experiences and deviant behaviors among Chinese rural emerging adults: the role of social support

**DOI:** 10.1186/s12889-022-14691-8

**Published:** 2022-12-21

**Authors:** Yiqing Wang, Shuang Ma, Ling Jiang, Qinian Chen, Jing Guo, Huan He, Pengyang Li, Tianjiao Gao, Xiaohua Wang

**Affiliations:** 1 School of Social Development and Public Policy, Beijing Normal University, No.19 Xin Jie Kou Wai Street, 100875 Beijing, People’s Republic of China; 2 School of Management, Beijing University of Chinese Medicine, Beijing, 100029 People’s Republic of China; 3 School of Public Health, Peking University, Beijing, 100191 People’s Republic of China; 4 School of Public Administration, Southwestern University of Finance and Economics, 555 Liutai Ave, Tongbo, Chengdu, Sichuan 611130 People’s Republic of China

**Keywords:** Adverse childhood experiences, Deviant behaviors, Social support, Emerging adults

## Abstract

**Background:**

ACEs hurt subsequent physical and mental health outcomes. However, still little has been known about the rate of ACEs among rural Chinese emerging adults and the different buffering effects of the three types of social support on different kinds of ACEs. This study described the rate of ACEs among Chinese rural emerging adults, examined the relationship between ACEs and deviant behaviors, and tested the moderating effect of three different sources of perceived social support on this relationship. We hope these results will be helpful in further interventions.

**Methods:**

We used the second wave of a longitudinal survey that included ACEs variables in 2018. A total of 1031 emerging adults aged 18 to 25 participated in the current study.

**Results:**

we found that (1) the rate of abuse, neglect, and household dysfunction was 10.0, 30.0, and 24.9%, respectively among Chinese rural emerging adults; (2) abuse and household dysfunction experience were significantly and positively associated with deviant behaviors; (3) friend support moderated the relationship between three types of ACEs and deviant behaviors. Other support moderated the relationship between abuse/household dysfunction and deviant behaviors.

**Conclusions:**

ACEs could increase the risk of deviant behaviors. Perceived friend support could reduce the negative effect of three types of ACEs. Other support could reduce the negative impact of abuse and household dysfunction. These results suggest that reducing ACEs to make children’s family environments safer and enhancing social support for emerging adults from rural areas are beneficial, which could prevent or reduce their deviant behaviors.

## Introduction

Adverse childhood experiences (ACEs) are stressful events, including child abuse (sexual, physical and emotional), neglect (physical and emotional), and various forms of household dysfunction (i.e., mental illness in the household and parental divorce) experienced before the child is 18 years old [1, 2]. ACEs have become an important public health concern, arousing widespread attention from researchers and the public [3]. A review study reported that the percentage of participants with at least one ACE was ranged from 33 to 88% among different people and different countries [3]. In China, the first known ACE study was conducted among 2073 Chinese medical college students in Anhui province, which found that 68.9% respondents reported at least one ACE [4]. Recent ACEs studies suggested that 66–75% of participants reported at least one ACE and ACEs were related to drinking, smoking, chronic diseases, and mental health (depression, anxiety, suicidality, etc.) [5–7]. However, still little has been known about the rate of ACEs among rural Chinese emerging adults. In Chinese rural areas, many parents migrate from rural to urban to seek better work opportunities and left their children in their hometowns [8]. Parental absence resulted in inadequate care and support for their children, which means more likely neglect [9]. A study among 1894 Chinese students found that rural students had more emotional abuse, sexual abuse, emotional neglect, and physical neglect than urban students [10]. Thus, ACEs in rural areas should be paid more attention and whether these ACEs could lead to more deviant behaviors should be further examined, which helps increase awareness of ACEs. In addition, some emerging adults from rural areas chose to work or get married instead of continuing higher education like most urban youth. Lower education and lack of family resources may lead them more vulnerable to negative development. Thus, focusing on Chinese rural emerging adults will be helpful in their development.

Emerging adults refers to the youth aged 18–25 [11]. Based on the Arnett’s theory of emerging adulthood, emerging adults have five distinctive characteristics:identity explorations, instability, self-focus, feeling in-between and a sense of possibilities [12]. They try out various life possibilities, make lots of explorations in work and marriage, etc., which make them more vulnerable to deviant behaviors. The program Monitoring the Future (MTF) suggested that throughout the lifespan, the annual and 30-day prevalence of using any illicit drug are highest in their early 20s [13, 14]. And ACEs could increase their risk of illicit drug use, binge drinking, gang involvement and crime [15, 16]. Thus, investigating deviant behaviors and the impact of ACEs on it among Chinese rural emerging adults could help in preventions and interventions.

### The relationship between ACEs and deviant behaviors

Many previous studies investigated the relationship between ACEs and multiple deviant behaviors including smoking, drinking, substance use, etc. A study drawing data from the National Longitudinal Survey of Youth (NLSY79) investigated early ACEs and smoking in adulthood and found significant associations between childhood physical abuse, household alcohol abuse, and household mental illness and ever smoked, and these results were replicated in different subgroups [17]. The results also from NLSY79 suggested that ACEs were significantly associated with childhood antisocial behavior, adolescent delinquent behavior, and violent crime victimization [18]. Another large study examined the relationship between early ACEs and behaviors at 9 and found that early ACEs could lead to more externalizing problems (aggressive behavior and rule-breaking) at 9 [19]. A recent review, covering 34 previous studies found that ACEs significantly predicted alcohol use (binge drinking, problem drinking, alcoholism, etc.), tobacco use, marijuana use, and other illicit drug use (cocaine, ecstasy, heroin, etc.) among young adults [20]. Another review study only focused on university students also found a significant relationship between ACEs and multiple deviant behaviors such as alcohol, tobacco, marijuana, and illicit drug consumption [21]. However, most previous studies focus on severe behaviors and investigate just one kind of behavior in each study. According to the *Diagnostic and Statistical Manual of Mental Disorders, fifth edition (DSM-5)*, deviant behaviors included aggressive behaviors (robbery, fighting, injuring others, etc.), fraud or theft, vandalism, and serious violations of rules (staying out late at night, often skipping school). And few studies investigate this relationship among Chinese rural emerging adults. This study will fill this gap.

### The role of social support

Barrera proposed two major types of social support-perceived (subjective) and received (objective) support [22]. And perceived support has been the most commonly assessed construct of social support [23, 24]. In addition, a recent study found that perceived support had a greater effect on depressive symptoms than received support, and the relationship between received support and depressive symptoms was fully mediated by perceived support [25]. Thus, perceived support is more important for individuals. Perceived social support was defined as the perception of his/her situation like how many available resources to cope with stress [25].

The stress-buffering model suggested two processes of social support buffering the negative effect of stress. First, social support could reduce stress by attenuating a stress appraisal response. An individual with a higher perception of available support could appraise lower harm of the stress and a higher ability to cope with the stress. Second, social support could eliminate stress by reducing the stress reaction or influencing physiological processes (e.g., perceiving less importance of the stress) [26]. For individuals with ACEs, adequate social support could make them perceive more resources to cope with the stress or reduce stress reaction (e.g., problem drinking) to the perceived stress, which means fewer deviant behaviors. Social support is also conceptualized as coping assistance to buffer the negative effect of stressful events by facilitating cognitive and emotional processing, thus reducing intense undesirable feelings [27]. Thus, social support was an important protect factor for individuals.

Empirical studies often focus on overall social support and found that it could reduce the negative impact of ACEs [28, 29]. However, perceived social support is derived from different sources, such as family, friends, and significant others [30]. Some previous studies investigated the different effects of three sources of social support. For example, Alsubaie et al. found that family and friend support predicted quality of life (psychological). Significant others and friends predicted quality of life (social relationships) [31]. Another study investigated the effects of support from family, friends, and romantic partners among emerging adults and found that only family support could buffer the effect of stress on depression [32]. These findings indicated different supports may function differently. Currently, few studies investigate the different buffering effects of the three types of social support on different kinds of ACEs. Thus, what kind of social support can mitigate the negative impact of ACEs still needs to be further examined, which is helpful in targeted interventions.

### The present study

This study aims to examine 1) the rate of ACEs among Chinese rural emerging adults, and its relationship with deviant behaviors; and 2) the moderating effects of the three sources of social support on the relationship between ACEs and deviant behaviors.

## Method

### Participants

We used the second wave data of a longitudinal study to understand the lives and mental health of rural children conducted by researchers. In this survey, we investigated individuals’ mental health (e.g., depression, self-esteem), physical health, and environmental factors (e.g., family environment, neighborhood environment), which aims to understand their status and further promote their overall development. In the initial survey in 2008, we first randomly selected two townships from the selected Zhenping County of Henan Province. Then, according to the distance from the primary school to the center of its county, we selected four primary schools in each township. Because there is only one junior high school in each township, the junior high schools in selected townships were all included. All students from grades 3–6 in eight primary schools and grades 7–9 in two junior high schools were invited to join this study. Excluding those who were reluctant to participate, other students participated in the current study. Eventually, 2067 rural students from grade 3 to grade 9 were included. They completed questionnaires after giving consent.

Between 2016 and 2018, 1031 (49.9%) participates were followed up, excluding the individuals who are reluctant to participate. They completed the two waves. ACEs and social support have been assessed in the second wave; thus, the second wave of data was used in this study. According to the definition of emerging adulthood, 162 participants under age 18 were excluded. According to the answers of deviation behavior scale, 15 invalid questionnaires were deleted. and 855 respondents were finally included in the current study (mean age = 20.90 years; SD = 1.86 years).

Data were collected in a variety of ways, since the subjects were in different schools or different workplaces. The project leader first contacted the teachers of these subjects, who went to the villages to collect the children’s current contact information, and then trained college and postgraduate students majoring in social sciences to contact the subjects through Tencent QQ (an instant messaging software), emails, telephone calls or other ways. The data were collected through the collection platform of the basic education quality inspection center of the Ministry of Education. At the same time, the snowballing method was used to find the contact information of other subjects who joined the survey in 2008. Moreover, considering that these subjects will go back to their hometown during the Spring Festival (Chinese New Year), we recruited questionnaire interviewers in their hometown and interviews were conducted by questionnaire interviewers when the subjects went back to their hometowns during the Spring Festival. All participants were informed of the aim of the survey and provided informed consent before they completed the questionnaire.

In the process of collecting data, we took the following quality control measures: 1) we trained questionnaire interviewers before collecting questionnaires to ensure that they understand the questionnaire and the process of collecting questionnaires thoroughly; 2) The interviewers are required to check the questionnaires after participants completing to avoid invalid questionnaires, such as the incomplete questionnaires.

### Measurements

**Deviant behaviors** were measured using a self-designed scale based on the DSM-5 [33] and Multiple Problem Behavior Index (MPBI) [34]. According to different social norms, we constructed scales for students (15 items) and working groups (10 items). Both two scales share 10 items, such as “illicit drug use”, “playing violent games”, and “stealing other people’s property”. There are 5 items (playing truant or skipping classes, smoking, drinking etc.) for student scale only. Each item was scored on a five-point scale (1 = *never* and 5 = *often*). Standardized t-scores with normal distributions were used to accommodate two versions of the deviant behavior measures for the different groups, with higher scores indicating more deviant behaviors. The Cronbach’s α of the student scale and work group scale were 0.785 and 0.864, respectively.

**Adverse childhood experiences (ACEs)** were assessed using the ACE questionnaire based on previous studies [35, 36]. This scale includes 14 items to measure the following three aspects: abuse (emotional, physical and sexual abuse), neglect (emotional and physical neglect), and household dysfunction (substance abuse, mental illness and incarceration of household member, parental separation/divorce). The questions of parental separation/divorce were binary (yes vs. no). The answers of the remaining items were “never,” “once or twice” “sometimes” “often” or “very often”. Because of the low rate of each response, dichotomous variables were created to reflect exposure to any ACE: responses of “never” were recoded “no” and “once or twice”, “often” or very “often” were recoded “yes” for these items. If respondents answered “yes” to at least one of the questions in a category, they were regarded as exposed to that category [37]. A recent study suggested that regardless of the ACEs measurement (prospectively or retrospectively), high ACE scores can identify groups of individuals at high-risk [38]. In this study, Cronbach’s alpha for this scale was 0.734. Confirmatory factor analysis indicated an acceptable fit (χ^2^/df = 7.672, *p* < 0.001, RMSEA = 0.088, NFI = 0.802, IFI = 0.823, CFI = 0.821).

**Translation process:** We used the forward-backward translation method [39]. First, two specialists independently translated the scale into Chinese and obtained the first version of this scale after discussing and correcting it. Second, two specialists in English translated the first Chinese draft into English. Finally, the expert group and researcher group compared and discussed the original scale, the first Chinese version, and the back-translated English scale and obtained the final scale.

**Perceived social support** was measured by 12 questions with a 7-point Likert-type scale (1 = strongly disagree, and 7 = strongly agree) using the Multidimensional Scale of Perceived Social Support (MSPSS) [40]. This scale includes three source types: family, friends, and others (colleagues and teachers). Every subscale includes four items and sum scores were used for each scale, with higher scores indicating higher levels of different support. The MSPSS has good internal reliability and construct validity among adolescents [41, 42]. The Cronbach’s α of the subscales of family support, friend support and other support were 0.882, 0.892 and 0.859 in this study, respectively.

**Confounding variables:** The control variables in the analyses were as follows: individual demographic variables (gender, age, marriage, educational level, worker or student), parents’ educational level (primary school or below, Junior high school, technical secondary school or above), family economic status, family migrant status (nonmigrant family, both-parent migration, father-only or mother-only, parents’ divorce or death), left-behind child. Parents’ educational level, family economic status, and family migrant status were measured in the baseline survey. Demographic variables and left-behind child were measured in the second wave.

Individual educational level was measured differently in the student group and nonstudent group. In the student group, respondents were asked “What type of school are you currently attending?” The answer was combined into two categories based on the sample distribution: vocational secondary school or high school and college or above. In the nonstudent group, the question “What was your education level when you were no longer in school?” was used. The answer was Junior high school or below/ High school/ College degree or above. Family economic status was measured by one question: “How do you think your family’s economic situation is?” The answer was rich, general or poor.

### Statistics

All data were analyzed using SPSS (Version 23.0) [43]. Because there are few missing values (the rate of missing values in each variable was less than 5%), we used the method - exclude cases listwise to handle the missing values when analyzing [44]. Descriptive analyses were used to show the sociodemographic of the sample and rate of ACEs. The ACE-deviant behavior relationship was assessed using multivariable linear regression. Finally, the moderating effects of the three types of social support on the relationship between ACEs and deviant behaviors were assessed using the Model 1 of the PROCESS macro [45].

## Results

### Descriptive analysis

55.1% of the respondents were male. Among these young adults, 33.9% were students, and 66.1% were not students. Their current educational level was described: 38.5% of the sample graduated from junior high school or below, and 28.2% were still in school or graduated from college or above. The percentage of participants who graduated from high school was 33.3%. In addition, 92.8% of these adults reported that their family economic status was general or poor. And 63.7% reported left behind experience: 24.1% had one parent away and 16.6% had both parents away when they are children. (Table 1).

**Table 1:** Descriptive Results of ACEs, Social Support and Demographics

Variable	N	M(SD)/%
Abuse	85	10.0%
emotional abuse	69	8.1%
physical abuse	47	5.6%
sexual abuse	18	2.1%
Neglect	262	30.6%
emotional neglect	75	8.8%
physical neglect	215	25.1%
Household dysfunction	211	24.9%
Parental separation or divorce	81	9.7%
Death in family	70	8.4%
Depressive or mentally ill household member	67	7.9%
suicide attempts member	48	5.7%
Substance abuse in home	42	5.0%
Incarcerated household member	30	3.6%
Number of ACEs		
0	463	54.2%
1	230	26.9%
2	72	8.4%
3	47	5.5%
≥ 4	43	5.0%
Deviant behaviors (at least one)	369	43.2%
Students	214	53.4%
Nonstudents	155	37.9%
Perceived social support		
Family support	853	5.25(1.35)
Friend support	853	5.30(1.24)
Other support	854	5.17(1.26)
Gender		
Male	471	55.1%
Female	384	44.9%
Age		
18–19	297	34.7%
20–21	315	36.8%
22–24	181	21.2%
> =24	62	7.3%
Family migrant status		
nonmigrant family	462	54.1%
both-parent migration	142	16.6%
father-only or mother-only	206	24.1%
Parents’ divorce or death	44	5.2%
Family economic status		
rich	61	7.2%
general	516	61.1%
poor	267	31.6%
Respondents’ educational level		
Junior high school or below	313	38.5%
High school	270	33.3%
College degree or above	229	28.2%
Marriage		
Married	190	22.6%
Unmarried	650	77.4%
Left-behind child		
No	310	36.3%
Yes	545	63.7%
Worker or student		
Student	290	33.9%
Worker	565	66.1%

*Note:* Denominator varies due to missing data for some variables

Regarding the rate of ACEs and deviant behaviors among Chinese rural emerging adults, 10.0% reported childhood abuse experience, with 8.1% emotional abuse, 5.6% physical abuse, and 2.1% sexual abuse. The rate of neglect was 30.6%, and of which 8.8% emotional neglect and 25.1% physical neglect. A total of 24.9% reported that they experienced household dysfunction (3.6–9.7% for each item). As for the number of ACEs, 45.8% of the participants reported at least one ACE. A total of 53.4% reported at least one deviant behavior in the student group and 37.9% in the nonstudent group. Moreover, the mean score was 5.25 (SD = 1.35) for family support, 5.30 (SD = 1.24) for friend support, and 5.17 (SD = 1.26) for other support, indicating that these young adults had a relative high level of social support (the score ranges from 1 to 7). (Table 1).

Regarding the rate of different deviant behaviors among Chinese rural emerging adults, we presented the results in Table 2. In the student group, the first three prevalent behaviors were “playing truant or skipping classes” “drinking” and “smoking”. In the working group, the first three prevalent behaviors were “going to pornographic venues” “gambling with others” and “playing violent games”.

**Table 2:** The rate of different deviant behaviors

Behaviors	Student group (*N* = 290)		Working group (*N* = 565)	
	N	%	N	%
Playing truant or skipping classes	93	33.90%		
Smoking	32	11.70%		
Drinking	88	32.10%		
Staying out all night without permission	32	11.70%		
Get away from home	7	2.60%		
Going to pornographic venues	16	5.50%	144	25.50%
Trying drugs secretly	3	1.00%	10	1.80%
Playing violent games	31	10.70%	99	17.60%
Gambling with others	20	6.90%	108	19.10%
Carrying knives or other weapons	9	3.10%	29	5.20%
Stealing others’ property	7	2.40%	12	2.10%
Fighting with others	15	5.20%	59	10.40%
Participating in a gang, and gang activities	5	1.70%	18	3.20%
Intimidating and extorting other people’s property	4	1.40%	10	1.80%
Destroying public property or other people’s property without reason	9	3.10%	18	3.20%

### The relationship between ACEs and deviant behaviors

As presented in Table 3, we investigated the relationship between ACEs and deviant behaviors by multivariable linear regression analysis and found that after adjusting for confounding variables, abuse (*β* = 0.094，*p* < 0.01) and household dysfunction (*β* = 0.203，*p* < 0.001) significantly predicted deviant behaviors. Adults who experienced abuse and household dysfunction having higher deviant behavior scores.

**Table 3:** Adverse Childhood Experiences Associated with Deviant Behaviors

	Deviant behavior		*t*	(95% CI)
	*B(SE)*	*β*		
Abuse (No)				
Yes	3.035 (1.149)	0.094	2.641^**^	[0.779, 5.292]
Neglect (No)				
Yes	0.743 (0.729)	0.035	1.020	[−0.688, 2.175]
Household dysfunction (No)				
Yes	4.529 (0.825)	0.203	5.487^***^	[2.909, 6.150]
Gender				
Male	5.001 (0.660)	0.261	7.580^***^	[3.706, 6.297]
Age	−0.400 (0.217)	−0.078	−1.840	[− 0.827, 0.027]
Family migrant status (nonmigrant family)				
both-parent migration	1.655 (0.947)	0.064	1.749	[−0.203, 3.513]
father-only or mother-only	0.123 (0.814)	0.006	0.152	[−1.474, 1.720]
Parents’ divorce or death	−3.508 (1.563)	−0.080	−2.245^*^	[−6.575, − 0.440]
Family economic status(general)				
rich	1.440 (1.277)	0.039	1.128	[−1.067, 3.946]
poor	−0.334 (0.716)	−0.016	− 0.466	[−1.739, 1.071]
Parental educational level (Junior high school)				
Primary school or below	0.812 (0.762)	0.038	1.065	[−0.685, 2.309]
Technical secondary school or above	2.793 (0.889)	0.111	3.141^**^	[1.048, 4.538]
Respondents’ educational level (High school)				
Junior high school or below	0.350 (0.831)	0.018	0.421	[−1.282, 1.982]
College degree or above	2.215 (0.990)	0.105	2.237^*^	[0.271, 4.159]
Marriage (Unmarried)				
Married	2.036 (0.902)	0.089	2.257^*^	[0.265, 3.808]
Left-behind child (No)				
Yes	0.122 (0.714)	0.006	0.172	[−1.278, 1.523]
Worker or student (Worker)				
student	0.336 (1.014)	0.017	0.331	[−1.655, 2.328]
Constant	51.727			[42.687, 60.768]
Observations	782			
R square	0.162			
Adjusted R square	0.144			
F	8.709^***^			

*Note: ß* beta, *(SE)* Standard error, *CI* 95% Confidence interval

^***^p < 0.001, ^**^p < 0.01, ^*^p < 0.05

### The moderating role of perceived social support

**Fig. 1:**
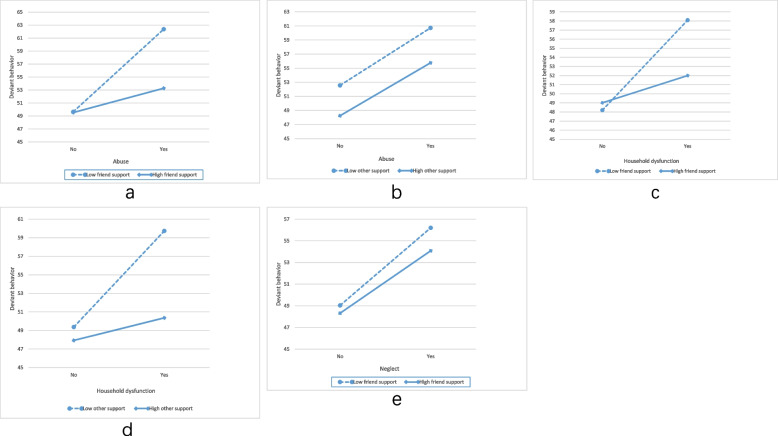
The Interaction Effect of ACEs and Social Support

The moderation model of three types of social support is presented in Table 4. We conduct nine models to examine the moderating effects of three types of social support using Model 1 of the PROCESS macro [45]. As model 1 an example, we entered deviant behaviors as the dependent variable, abuse as an independent variable, and family support as moderating variable. Gender, age, family migrant status, family economic status, parental educational level, respondents’ educational level, marriage, left-behind children, worker or student were covariates in each model. We found significant moderating effects of friend support and other support on the relationship between abuse/ household dysfunction and deviant behavior (the 95% confidence interval did not include 0), while no significant moderating effect of family support on these relationships. That is, youth with abuse or household dysfunction experience but who had high levels of perceived friend support or other support had less deviant behavior than those with low levels of friend support or other support (Fig. 1, a-d).

**Table 4:** The Moderating Effects of Social Support

	Moderating effects	Coeffect	SE	95% CI		*R*^*2*^*-chng*
				Lower	Higher	
Model 1	Abuse * Family support	−0.916	0.778	−2.443	0.611	0.0015
Model 2	Abuse * Friend support	−2.487	0.850	−4.155	−0.818	0.0096^**^
Model 3	Abuse * Other support	−1.767	0.809	−3.355	−0.179	0.0053^*^
Model 4	Neglect * Family support	−0.347	0.512	−1.353	0.658	0.0005
Model 5	Neglect * Friend support	−1.175	0.573	−2.300	−0.050	0.0048^*^
Model 6	Neglect * Other support	−0.810	0.567	−1.922	0.302	0.0023
Model 7	Household dysfunction * Family support	−0.937	0.543	−2.003	0.130	0.0032
Model 8	Household dysfunction * Friend support	−1.820	0.579	−2.955	−0.684	0.0108^**^
Model 9	Household dysfunction * Other support	−1.356	0.573	−2.481	−0.231	0.0061^*^

*Note: SE* Standard error, *CI* 95% Confidence interval

^***^ p < 0.001, ^**^ p < 0.01, ^*^ p < 0.05

*R*^*2*^*-chng:* R-square increase due to interaction. If this increment was significant, the interaction effect was significant

Gender, age, family migrant status, family economic status, parental educational level, respondents’ educational level, marriage, left-behind children, worker or student were covariates in each model

We also found that friend support plays a moderating role in the relationship between neglect and deviant behaviors. Youth who experienced childhood neglect but who perceived high levels of friend support had less deviant behavior than those with low levels of friend support or other support (Fig. 1, e).

## Discussion

This study described the rate of ACEs, examined the relationship between ACEs and deviant behaviors, and examined the moderating effects of three types of perceived social support on this relationship among Chinese rural emerging adults. We found that the rate of abuse was 10.0, 30.6% for neglect and 24.9% for household dysfunction. Exposure to abuse and household dysfunction significantly and positively predicted deviant behaviors. In addition, perceived friend support moderated the relationship between three types of ACEs and deviant behaviors. Other support moderated the relationship between abuse/household dysfunction and deviant behaviors. These findings support that reducing ACEs to make children’s family environments safer and enhancing social support for emerging adults from rural areas, which could prevent or reduce their deviant behaviors targeted.

### The rate of ACEs and deviant behaviors

The rate was 10.0% for abuse, 30.6% for neglect and 24.9% for household dysfunction among Chinese rural emerging adults. These findings parallel the results of a recent meta-analysis study. This study covered 337 previous studies and found that the prevalence was 9.2–33.4% for emotional abuse, 6.7–18.9% for physical abuse, 2.6–18.2% for sexual abuse and 6.6–47.2% for child neglect in different countries [46]. A nationally representative survey among people with lower socioeconomic status suggested that emotional abuse (34.42%) was the most common one and the prevalence was 17.9% for physical abuse, 11.6% for sexual abuse, 7.9–27.6% for household dysfunction [47]. In China, the rate of emotional neglect was 51.3, 34.9% for physical neglect, 52.5% for emotional abuse, 35.9% for physical abuse, and 1.9–8.5% for different items of household dysfunction among junior high school students in Shanghai [48]. Among rural Chinese young adults from three different provinces, the rate was 4.7% for physical neglect, 8.2% for emotional neglect, 6.0% for emotional abuse, 52.3% for physical abuse,10.6% for sexual abuse and 8.0–43.2% for household dysfunction [7]. ACEs rate was estimated differently in different studies, due at least partly to different measurements and sample populations. This finding increased awareness of ACEs among Chinese rural emerging adults.

We found that the first three prevalent behaviors among the student group were “playing truant or skipping classes” “drinking” and “smoking”. In the working group, the first three prevalent behaviors were “going to pornographic venues” “gambling with others” and “playing violent games”. The rate was different in different studies. Drinking and smoking were common behaviors among emerging adults. A recent study investigating emerging and young Chinese adults (aged 18–34) in Wuhan province found that the rate of drinking alcohol was 45.84% [49]. A meta-analysis, covering 41 studies found that the pooled rate of smoking among Chinese university students was 13.8% [50]. Yu et al. investigated the behaviors of university students using the national representative sample and found that the rate of drinking and smoking were 3.62 and 5.57% [51]. Regarding the rate of gambling, a review study about Hong Kong youth suggested that the rate of gambling rang 28–70%, which was comparable to the global findings [52]. A national study in Australia found that 56.9% of participants gambling at least one type of [53]. These differences in rate may be due to different areas, samples, and measurements. In addition, few studies examined the rate of “going to pornographic venues” and “playing violent games”, but studies have proved the negative effects of violent games [54]. Thus, reducing deviant behaviors is also important.

However, it should be noted that the rate was drawn from the sample from one province in China. Thus, it should be cautious about its generalization of it.

### The impact of ACEs on deviant behaviors

We found that after accounting for sociodemographic controls, there was a strong association between ACEs and deviant behaviors. Adults who had abuse and household dysfunction experience reported more deviant behaviors than those without ACEs. This result is consistent with the findings of a recent systematic review and meta-analysis which found that ACEs were strongly associated with negative developmental outcomes, including different problem behaviors [3, 55]. However, the difference with previous studies was that we focus on multiple problem behaviors. That is, adults with ACEs are more likely to experience multiple problem behaviors. This study also supported the developmental psychopathology perspectives and the strain theory. Developmental psychopathology perspectives suggested that children who experience abuse and neglect are more likely to experience inadequate maturation and adaptation later in life, thus lead to developmental risks [56], such as deviant behaviors. As the strain theory suggested, deviant behavior could be acted as a way of coping with environment stress. Individuals who had ACEs are hypothesized lack of resources to achieve socially accepted goals, which lead to strain. This strain may compel an individual to engage in deviant behaviors to attain the positively valued goal of happiness [57].

### The moderating role of perceived social support

We found that perceived friend support moderated the relationship between three types of ACEs and deviant behaviors. Other support moderated the relationship between abuse/household dysfunction and deviant behaviors. That is, deviant behaviors were lower in people who experienced any type of ACEs if they had high levels of friend support. With high levels of other support, deviant behaviors were lower in people with abuse and household dysfunction. These results supported the stress buffering theory [26] and protect factor model which suggested that a protect factor could compensate for the negative effect of a risk factor [58] The mechanisms of this moderating effect could be explained as follows: Cicchetti proposed the ecological-transactional model, which asserts that the individual’s protective factors and risk factors jointly act on the individual’s development. When the protective factors exceed the risk factors, the individual develops well [59]. Thus, if people with adverse family experiences could perceive good support from friends or other interpersonal relationships, they could recover from the impact of adverse events. Similar effects have been reported in previous empirical studies [60, 61]. However, unlike previous studies focusing on overall social support, the current study distinguished the different effect of three type of social support, indicating different interventions for people with different ACEs. These findings highlight the importance of friend support for people with any type of ACEs and the importance of other support for people with abuse and household dysfunction experiences. Thus, these results provided evidence for targeted intervention.

In addition, perceived family support does not have a moderating effect on the relationship between the three types of ACEs and deviant behaviors. These findings could be explained as follows: Household dysfunction refers to the events that parental divorce or separation, household substance abuse or incarcerated household member, neglect refers to not having enough food for children, or not caring about the behaviors of children, while abuse refers to hitting children or sexual assault. These events were all from family members and directly damage children physically or emotionally, which indicated that parents or caregivers did not provide support for their children. That is, ACEs mean less or no family support. In this sample, we found that abuse, neglect, and household dysfunction affected deviant behaviors by destroying family support (it was not shown in this study and needs a further longitudinal study to examine it). Thus, family support could not reduce the negative effect of ACEs.

### Limitations and implications

This study produced some findings about the relationships between ACEs, deviant behaviors, and perceived social support among rural Chinese emerging adults. However, there are several limitations. First, we only used the second wave of a longitudinal survey, which means this is a cross-sectional study, thus no causal relationships could be confirmed between ACEs and deviant behaviors. Longitudinal designs were needed to examine the causal relationship and mediating effect of social support in the future. Second, the retrospective measurement of ACEs may lead to the recall bias of the subjects and the deviation of the measurement results. Moreover, an individual is affected by various factors in the process of growth. These potential confounding factors were not considered or controlled, possibly leading to bias in the analyses. Future study should include more confounding. Third, although the ACE scale has been widely used in foreign (e.g., Western) studies, it is still rarely used in Chinese culture, and some items of the scale may be different from the actual situation in China. In future studies, the localized revision of the ACE scale can be carried out to make it more applicable to Chinese culture. Fourth, the respondents in this study include students and nonstudents, who have different characteristics, so future studies can explore the relationship between ACE and deviant behavior in the two groups. Fifth, the participants in this study were recruited from only one area; thus, it is necessary to be cautious about the generalization of these findings. In the future study, we will investigate different samples or national-based samples.

Despite these limitations, the findings of the current study have significant practical implications. Our findings increase awareness of ACEs among Chinese rural emerging adults and support the idea that reducing adverse childhood experiences can improve children’s family environment for development. In addition, in terms of adults with ACEs, it is crucial to enhancing social support from friends and others (colleagues or teachers) to protect them from adverse effects, thus, improve their mental health.

## Conclusion

The rate of childhood abuse, neglect, and household dysfunction were 10.0, 30.6, and 24.9% respectively among Chinese rural emerging adults. Individuals who had ACEs had an increased risk of deviant behaviors. Perceived friend support moderated the relationship between three types of ACEs and deviant behaviors. Other support moderated the relationship between abuse/household dysfunction and deviant behaviors. Family support moderated the relationship between household dysfunction and deviant behaviors. These findings suggested that reducing ACEs to make children’s family environments safer and enhancing targeted social support for emerging adults with different ACEs from rural areas are important and necessary, which could prevent or reduce their deviant behaviors.

## Data Availability

The raw data, analysis code, and materials used in this study are not publicly available due to protect the privacy and confidentiality of participants in this study but are available upon reasonable request to the corresponding author.
